# Restricted Gene Flow for *Gadus macrocephalus* from Yellow Sea Based on Microsatellite Markers: Geographic Block of Tsushima Current

**DOI:** 10.3390/ijms17040467

**Published:** 2016-03-29

**Authors:** Na Song, Ming Liu, Takashi Yanagimoto, Yasunori Sakurai, Zhi-Qiang Han, Tian-Xiang Gao

**Affiliations:** 1 Fisheries College, Ocean University of China, Qingdao 266003, China; songna624@163.com (N.S.); shelleyliuming@hotmail.com (M.L.); 2 National Research Institute of Fisheries Science, Fisheries Research Agency, Yokohama 220-6115, Japan; yanagimo@affrc.go.jp; 3 Graduate School of Fisheries Sciences, Hokkaido University, Hokkaido 041-8611, Japan; sakurai@fish.hokudai.ac.jp; 4 Fishery College, Zhejiang Ocean University, Zhoushan 316022, China; d6339124@163.com

**Keywords:** Pacific cod, microsatellite, genetic diversity, population genetic structure

## Abstract

The Pacific cod *Gadus macrocephalus* is a demersal, economically important fish in the family Gadidae. Population genetic differentiation of Pacific cod was examined across its northwestern Pacific range by screening variation of eight microsatellite loci in the present study. All four populations exhibited high genetic diversity. Pairwise fixation index (*F*_st_) suggested a moderate to high level of genetic differentiation among populations. Population of the Yellow Sea (YS) showed higher genetic difference compared to the other three populations based on the results of pairwise *F*_st_, three-dimensional factorial correspondence analysis (3D-FCA) and STRUCTURE, which implied restricted gene flow among them. Wilcoxon signed rank tests suggested no significant heterozygosity excess and no recent genetic bottleneck events were detected. Microsatellite DNA is an effective molecular marker for detecting the phylogeographic pattern of Pacific cod, and these Pacific cod populations should be three management units.

## 1. Introduction

The Pacific cod *Gadus macrocephalus* is a demersal, economically important fish in the family Gadidae. It is mainly distributed in the North Pacific, from the Yellow Sea of China through the Sea of Japan, Okhotsk and Bering Seas to California in the eastern North Pacific [1,2]. As an important cold-water species distributed in the Yellow Sea, the distribution of Pacific cod was closely related with the Yellow Sea Cold Water Mass [3]. Low temperature provides a suitable environment for the survival of Pacific cod and the Tsushima Current may delineate the distribution borders. The life history of Pacific cod comprises demersal eggs, pelagic larvae and demersal adults [1,4,5]. The literature indicates that this species could migrate for much longer distances than expected based on tag-recapture data, and it can explain genetic homogeneity in Pacific cod over broad areas of the North Pacific [2,6]. Could the Tsushima Current be a dispersal barrier of Pacific cod from the Yellow Sea? In the present study we want to study how the Tsushima Current affects the dispersal of cold-water fish by checking the genetic structure of Pacific cod.

Studies on population genetics have attracted much attention because such information is important for understanding the pattern of biogeography and sustainable utilization of fishery resources [7]. If we ignore the population genetic structure, over-fishing may lead to the extinction of the population with fewer individuals and reduce the genetic diversity of this species [8,9,10]. Until now, some genetic studies about the Pacific cod based on allozyme, mitochondrial DNA and microsatellite DNA have been conducted; however, their results suggested different genetic diversity and structures. Grant *et al.* [2] detected two major genetic groups (a western North Pacific Ocean group and an eastern North Pacific group) by an allozyme marker. Moreover, small but significant genetic differentiation was detected among northwestern Pacific populations based on mitochondrial DNA [11]. However, Cunningham *et al.* [12] examined the genetic population structure of Pacific cod by screening for variation at 11 microsatellite DNA loci, and higher genetic diversity across the northeastern Pacific was found and genetic divergence highly correlated with geographic distance was suggested. A strong genetic discontinuity between northwestern and northeastern Pacific populations was detected by Canino *et al.* and the current distribution pattern represents groups previously isolated during glaciations that are now in secondary contact [13].

These studies suggested different sensitivities of three molecular markers for detecting the genetic structure and diversity of the Pacific cod. Compared with mitochondrial DNA and allozyme markers, microsatellite DNA is a type of nuclear genetic marker that has been proven to be more sensitive in detecting population genetic structure [14,15,16]. In the previous studies, only samples across the northeastern Pacific range were collected [12], but no northwestern Pacific populations were employed. In the present study, we collected fish samples from the Yellow Sea, the Sea of Japan, the Okhotsk Sea and Eastern Hokkaido to conduct the genetic analysis and describe the genetic situation of Pacific cod by comparing these results with the results of Cunningham *et al.* [12]. The results of the present study will reveal the genetic structure and diversity of this economically important species and provide vital information for sustainable exploitation and management of natural populations.

## 2. Results

Clear and unambiguous bands were identified by denaturing polyacrylamide gel electrophoresis. All eight microsatellite markers were highly polymorphic and the number of alleles per locus varied from eight to 24 (Table 1). The number of alleles (*A*), observed heterozygosity (*H*o), expected heterozygosity (*H*e) and polymorphism information content (PIC) are shown in Table 1. The expected heterozygosity (*H*e) ranged from 0.496 (Gmo107, the Yellow Sea (YS)) to 0.957 (Gmo104, the Sea of Japan (SJ)), and the observed heterozygosity ranged from 0.435 (Gmo102, SJ) to 1.000 (Gmo37, Eastern Hokkaido (EH)). The PIC values ranged from 0.472–0.933, which suggested high genetic diversity of this species (PIC > 0.5) [17]. Five deviations from Hardy-Weinberg equilibrium tests were detected after Bonferroni correction: population EH at Gma107, population SJ at Gma102 and Gma104, population the Okhotsk Sea (OS) at Gmo08 and population YS at Gma107, respectively. The null alleles for these loci were also detected. Since the null alleles had a very low effect on the average genetic diversity of our data, we retained all loci for further study. The test for linkage disequilibrium for populations and loci showed a very low value of significant pairwise comparisons.

**Figure 1:**
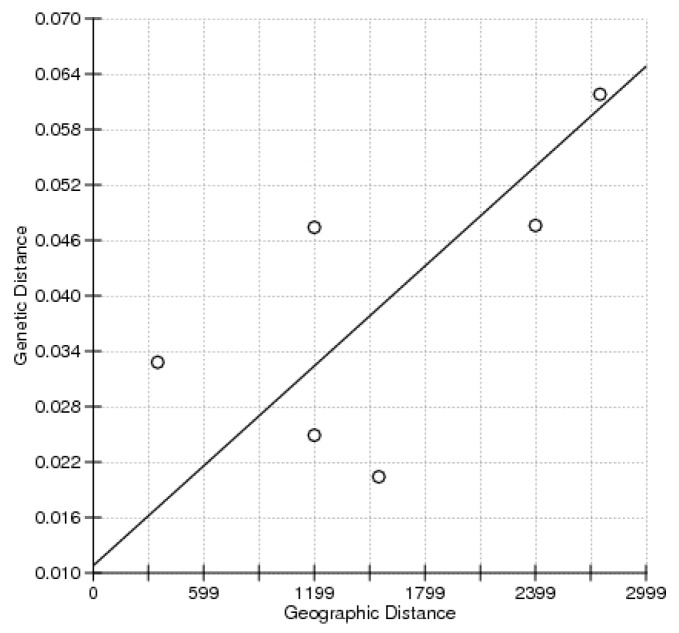
Plot of pairwise estimates of *F*_st_/(1 − *F*_st_) and geographic distance. The reduced major axis (RMA) regression line overlays the scatter plots.

**Figure 2:**
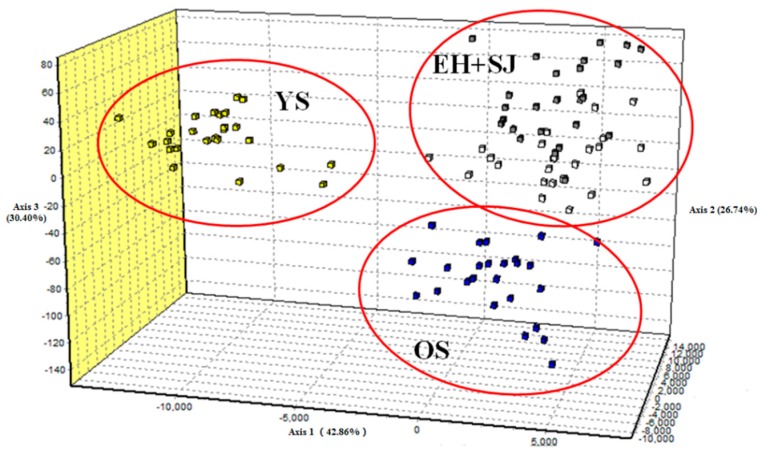
Three-dimensional factorial correspondence analysis (3D-FCA) showing the relationships among individuals of four populations based on eight microsatellite loci.

The values of *F*_st_ ranged from 0.0204–0.0618 and *p*-values were adjusted using the sequential Bonferroni procedure (Table 2). Populations SJ and OS showed the lowest genetic divergence (*F*_st_ = 0.0204; *p* < 0.05). Genetic differences between population YS and the other three populations were larger and statistically significant after sequential Bonferroni correction. The Mantel test indicated no significant relationship between pairwise estimates of *F*_st_/(1 − *F*_st_) and geographic distance (*r* = 0.6366; *p* = 0.132) (Figure 1). In order to further detect genetic structure, analysis of molecular variance (AMOVA) was performed under three patterns of gene pools. Most of the variances were found within populations for all three patterns. When two gene pools were tested, the values of *F*_CT_ were largest but not significant (*F*_CT_ = 0.035, *p* > 0.05) (Table 3). The first and second principal components explained 73.26% of the overall variation in the three-dimensional factorial correspondence analysis (3D-FCA), which separated all individuals into three groups (Figure 2).

**Figure 3:**
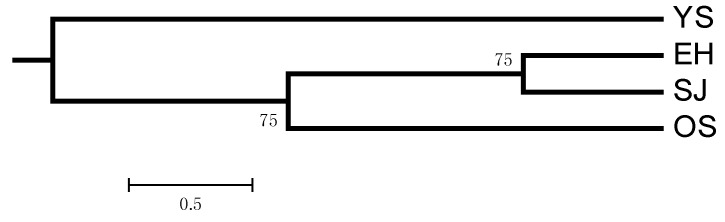
The unweighted pair-group method analysis (UPGMA) tree based on the (δμ)^2^ genetic distance of four Pacific cod populations. Results of bootstrapping (1000 replicates) are shown at nodes.

The (δμ)^2^ genetic distance was calculated according to the allele frequency and the results showed that population YS exhibited the larger genetic distance from the other three populations, which was in accordance with the result of the pairwise *F*_st_. However, the (δμ)^2^ genetic distance between populations SJ and EH was the smallest, not between populations SJ and OS. The topology of the unweighted pair-group method analysis (UPGMA) tree based on (δμ)^2^ genetic distance showed the relationship of the four populations more intuitively (Figure 3).

Wilcoxon signed rank tests under the three mutational models (the infinite allele model (IAM); stepwise mutation model (SMM); two-phase mutation model (TPM)) were not significant, which suggested that no significant heterozygosity excess was detected (Table 4). In addition, there is no evidence for a significant deviation from the normal l-shaped distribution of allele frequencies as expected for a stable population under mutation-drift equilibrium.

**Figure 4:**
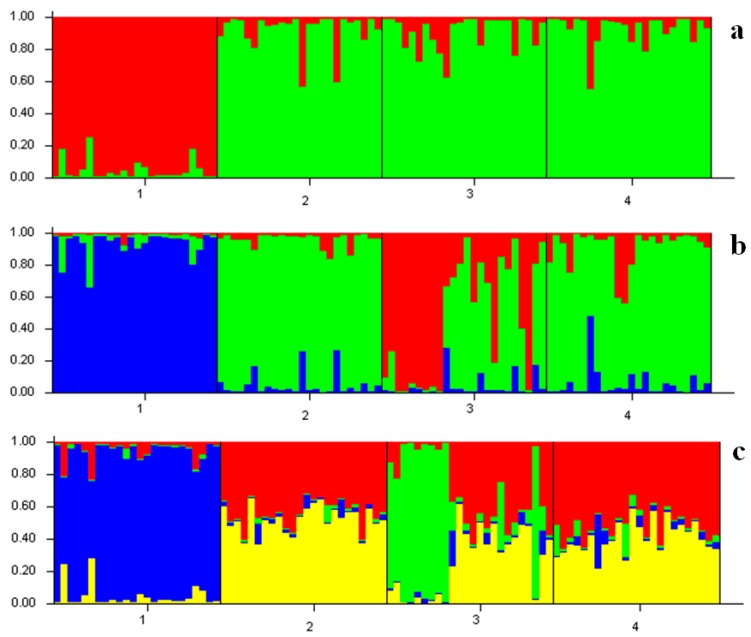
STRUCTURE bar plots from eight microsatellite loci for four populations of Pacific cod. Black lines separate individuals from different geographic areas. (**a**) *K* = 2; (**b**) *K* = 3; (**c**) *K* = 4; 1: YS; 2: EH; 3: OS and 4: SJ.

**Figure 5:**
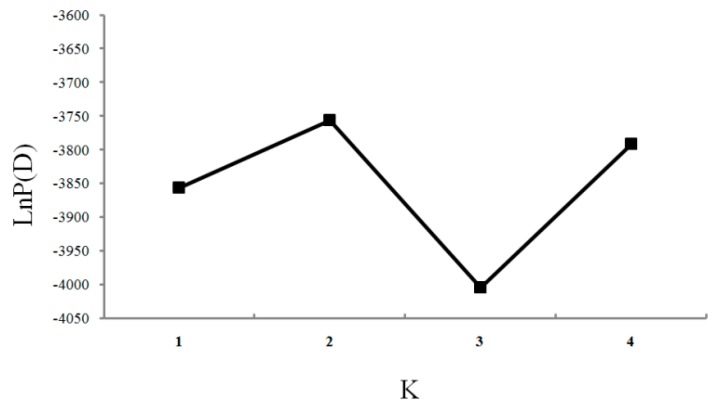
The model choice criterion ln*P*(*D*) for each *K* value and graphical results of the STRUCTURE analysis.

The Bayesian algorithm implemented in the program STRUCTURE indicated that the model with *K* = 2 explained the data in a satisfactory manner, as this model resulted in the highest Δ*K* value (Figure 4 and Figure 5). Two clusters were detected from the four populations. Population YS formed the first cluster with 94.9% of the sampled individuals, and 91.6%, 90.4%, 91.9% from the other respective populations contributed to the second cluster (Table 5).

## 3. Discussion

The results of the present study showed that there were higher genetic differences between population YS and the other three populations. The (δμ)^2^ genetic distance between population YS and the other populations was larger than other pairwise genetic distances, which was supported by the results of 3D-FCA and STRUCTURE. In the marine environment, currents can be critical for dispersal of marine fish, and then gene flow among populations may be influenced by marine currents [18,19]. Though tag-recapture data implied potential dispersal of Pacific cod, the Kuroshio Current and its branch, theTsushima Current, may block the gene exchange of Pacific cod because it is a cold-temperature fish species. The optimal spawning temperature for Pacific cod ranges from 0 to 13 °C, but the warm-water currents such as the Tsushima Warm Current may act as an exchange barrier for this species due to their higher seawater temperature [20]. Population YS is enclosed by land to the north and east, and by warm subtropical waters to the west and south [2,21]. The restricted gene flow between the Yellow Sea and the Sea of Japan was also supported based on allozyme markers by Gong *et al.* [22].

The strong genetic differentiation between populations from the Sea of Japan and the Okhotsk Sea determined with mitochondrial DNA by Liu *et al.* [11] was in contrast with the results of the present study. Shaw *et al.* [23] examined the population genetic structure of the Patagonian toothfish using mtDNA and nuclear DNA, and a similar situation was detected. He thought population patterns may reflect either genome population size effects or (putative) male-biased dispersal. The life history of Pacific cod comprises demersal eggs, pelagic larvae and demersal adults. Combined with the results of the present study and the life-history characteristics of Pacific cod, a smaller effective population size of mtDNA may enlarge the difference between populations and strong genetic differentiation was detected. In brief, a moderate to high level of genetic differentiation was detected for Pacific cod in the present study, which suggested the high sensitivity of the microsatellite marker. The Bayesian clustering analysis by STRUCTURE suggested the Okhotsk Sea populations were genetically distinct from the populations of Japanese coastal waters when *K* = 3 was performed, which was supported by the results of (δμ)^2^ genetic distance and 3D-FCA. The topology of the UPGMA tree based on (δμ)^2^ genetic distance showed that populations from the Japanese coast clustered with each other at first, and then with population OS. The pairwise *F*_st_ was calculated based on the haplotype frequency, and genetic distance was based on the difference of alleles. There are alleles that may cause this difference between the two methods.

The conservation of genetic diversity is very important for the long-term interest of any species [24]. Studies showed that population size must be kept at a certain level because loss of heterozygosity could have a deleterious effect on population fitness [25]. In the present study, the expected heterozygosity (*H*e) of Pacific cod was higher than the average values of marine fish [26], and most of the values of polymorphism information content (PIC) were more than 0.8, which suggested that high genetic diversity was detected based on the microsatellite marker. The level of genetic diversity was in accordance with the genetic examination of this species across its northeastern Pacific range by Cunningham *et al.* [12]. Moreover, four populations indicated a similar genetic diversity and did not show any geographical trends. Plenty of microsatellite analysis showed that marine fish usually exhibit high genetic diversity, which may be attributed to the high mutation rate of microsatellites or the huge population size of marine fish [14,15,16,27].

However, low genetic diversity of this species was detected by allozyme and mitochondrial DNA, which was in contrast with our results and Cunningham *et al.* [12]. Especially very low nucleotide diversity was detected based on mitochondrial DNA by Liu *et al.* [11]. The allozyme marker may be easily affected by environmental factors as it is a protein level marker, and the mitochondrial DNA was maternally inherited. These two markers may depart from neutral because they are easily affected by selection pressure [28,29]. Previous studies showed that the common mutation rate of microsatellite loci was faster than that of the mtDNA control region [13,30,31]. The results of the present study proved that the microsatellite marker was more sensitive for detecting the population genetic diversity of Pacific cod.

In conclusion, microsatellite DNA is an effective molecular marker for detecting the phylogeographic pattern of Pacific cod. The results of the present study suggested these Pacific cod populations should be considered as three management units and population YS should be paid more attention because of its uniqueness.

## 4. Experimental Section

### 4.1. Fish Samples

**Figure 6:**
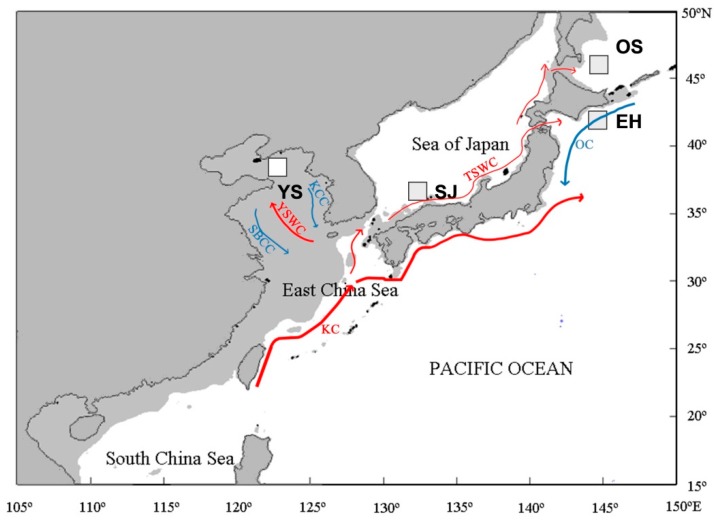
Sampling sites of Pacific cod in the present study. Shaded sea areas are continental shelves that would have been exposed to the air during periods of low sea-level. KC: Kuroshio Current; YSWC: Yellow Sea Warm Current; SBCC: Subei Coastal Current; KCC: Korean Coastal Current; TSWC: Tsushima Warm Current; OC: Oyashio current.

Samples from four locations across the northwestern Pacific Ocean were used in the present study (Figure 6, Table 6). A total of 96 individuals were collected during 2004–2005 and all individuals were identified on the basis of morphology, and a piece of muscle was taken from each individual and preserved in 95% ethanol or frozen for DNA extraction.

### 4.2. DNA Extraction and PCR Amplification

Genomic DNA was isolated from muscle tissue by proteinase K digestion followed by a standard phenol-chloroform method. Eight polymorphic microsatellite markers were employed for this study [32,33] (Table 7).

Polymerase chain reaction (PCR) was carried out in 10 μL volumes containing 0.5 U Taq DNA polymerase (Takara Co., Dalian, China), 100 ng template DNA, 0.15 μM of each forward and reverse primers, 0.2 mM each deoxy-ribonucleoside triphosphate (dNTPs), 10 mM Tris (pH 8.3), 50 mM KCl, 3.0 mM MgCl_2_. The PCR amplification was carried out in a thermal cycler (Biometra, Göttingen, Germany) under the following conditions: 5 min initial denaturation at 92 °C, and then followed by 25 cycles of 30 s at 92 °C, 30 s at annealing temperature 62–57 °C, 30 s at 72 °C and final extending for 30 min at 72 °C. We used 8% non-denaturing vertical polyacrylamide gel electrophoresis to separated PCR products and visualized by silver staining [34]. A sizing standard (100–300 base pairs) was run in the center and at both ends of each gel to calibrate allele size. Furthermore, a reference sample was run on each gel to ensure consistency in genotype scoring across runs.

### 4.3. Data Analysis

Deviations from the Hardy-Weinberg equilibrium (HWE) and linkage disequilibrium of each locus within each site were checked by GENEPOP 3.4 [35]. The number of alleles (*A*), observed heterozygosity (*H*o), expected heterozygosity (*H*e) and Polymorphism Information Content (PIC) were obtained by Microsoft Excel tools [17]. The null alleles in each population were checked by Micro-Checker 2.2.3 [36]. The values of pairwise *F*_st_ were calculated by FSTAT 2.9.3 [37]. Bottleneck 1.2.02 program [38] was used to detect the evidence of recent bottleneck events under three different mutation models: the infinite allele model (IAM), stepwise mutation model (SMM) and two-phase mutation model (TPM), where 95% single-step mutations and 5% multiple steps mutations with 1000 simulation iterations were set as recommended [39]. We also proposed a graphical descriptor of the shape of the allele frequency distribution (mode shift indicator) which could differentiate between bottlenecked and stable populations [40]. ARLEQUIN 3.1 was employed to assess the population structure by the analysis of molecular variance (AMOVA) [41], and AMOVA was carried out with different gene pools. In order to detect the suitable groups for AMOVA, *F*_st_ measures were subjected to multidimensional scaling (MDS) and plotted in two dimensions (applied using SPSS 11.0 (SPSS Inc., Chicago, IL, USA), data not shown). The relationship between genetic distances and geographic distances was assessed using Reduced Major Axis (RMA) regression and Mantel tests using IBDWS [42,43]. We calculated the (δμ)^2^ genetic distance by POPULATION 1.2 [44] and constructed the UPGMA tree based on the (δμ)^2^ genetic distance by MEGA 5 [45]. Three-dimensional factorial correspondence analysis (3D-FCA) was performed in GENETIX 4.05 to explore population divisions and relationships of Pacific cod, independent from prior knowledge of their relationships [46]. The possibility of cryptic population structure of Pacific cod was detected by STRUCTURE 2.2 [47]. Markov chain Monte Carlo (MCMC) consisted of 100,000 burn-in iterations followed by 1,000,000 iterations. The simulated *K* values ranged from 1 to 10 (total sites). Ten independent runs were implemented for each specific *K*-value in order to verify the consistency of the results. The *ad hoc* estimated likelihood of *K* (Δ*K*) was used to determine the most likely number of populations (*K*) based on the rate of change in the log probability of the data (Ln *P*(*D*)) [48].

## Figures and Tables

**Table 1:** Genetic diversity indices for Pacific cod.

Location	Locus									Average
	Parameters	Gma102	Gma104	Gma107	Gma108	Gma109	Gmo08	Gmo19	Gmo37	
The Yellow Sea (YS)	*A*	12	15	9	8	10	13	12	12	11.375 (2.264)
	*H*_E_	0.892	0.91	0.496	0.688	0.857	0.901	0.888	0.89	0.815 (0.148)
	*H*_O_	0.583	0.958	0.583	0.708	0.762	0.826	0.8	0.792	0.752 (0.126)
	PIC	0.861	0.883	0.472	0.634	0.817	0.871	0.853	0.858	0.781 (0.149)
Eastern Hokkaido (EH)	*A*	12	18	10	11	18	14	12	17	14 (3.251)
	*H*_E_	0.869	0.922	0.728	0.594	0.878	0.893	0.852	0.91	0.831 (0.113)
	*H*_O_	0.667	0.917	0.565	0.625	0.917	0.875	0.875	1	0.805 (0.161)
	PIC	0.834	0.896	0.692	0.56	0.847	0.864	0.82	0.881	0.799 (0.115)
The Okhotsk Sea (OS)	*A*	10	14	10	10	15	12	11	12	11.75 (1.909)
	*H*_E_	0.804	0.921	0.862	0.659	0.915	0.859	0.891	0.901	0.852 (0.087)
	*H*_O_	0.667	0.857	0.714	0.682	0.7	0.652	0.783	0.95	0.0751 (0.105)
	PIC	0.758	0.89	0.812	0.621	0.883	0.827	0.859	0.867	0.815 (0.089)
The Sea of Japan (SJ)	*A*	11	24	13	14	21	14	13	13	15.375 (4.565)
	*H*_E_	0.871	0.957	0.816	0.629	0.942	0.848	0.886	0.848	0.850 (0.101)
	*H*_O_	0.5	0.833	0.667	0.667	0.87	0.833	0.833	0.875	0.760 (0.134)
	PIC	0.836	0.933	0.78	0.602	0.917	0.814	0.854	0.817	0.819 (0.102)

*A*: allelic number; *H*_O_: observed heterozygosity; *H*_E_: expected heterozygosity; PIC: polymorphism information content.

**Table 2:** Pairwise *F*_st_ (below diagonal) and (δμ)^2^ distance (above diagonal) among populations of Pacific cod.

Population	YS	EH	OS	SJ
YS	–	4.1303	4.8946	5.7433
EH	0.0476 *	–	3.0133	1.1149
OS	0.0618 *	0.0328 *	–	1.7213
SJ	0.0474 *	0.0249 *	0.0204 *	–

* Significant after Bonferroni correction.

**Table 3:** The analysis of molecular variance of Pacific cod.

Gene Pools	Structure Tested	Variance (%)	*F* Statistics	*p*
One pool	(YS, SJ, OS, EH)	–	–	–
	Among populations	3.86	*F*_ST_ = 0.039	0.00
	Within populations	96.14	–	–
Two pools	(YS) *vs.* (SJ, OS, EH)	–	–	–
	Among groups	3.45	*F*_CT_ = 0.035	0.23
	Within groups	2.07	*F*_SC_ = 0.021	0.00
	Within populations	94.48	*F*_ST_ = 0.055	0.00
Three pools	(YS) *vs.* (SJ, EH) *vs.* (OS)	–	–	–
	Among groups	1.09	*F*_CT_ = 0.011	0.49
	Within groups	2.95	*F*_SC_ = 0.030	0.74
	Within populations	95.97	*F*_ST_ = 0.040	0.00

**Table 4:** Results of Wilcoxon’s heterozygosity excess test, the mode shift indicator for a genetic bottleneck in four Pacific cod populations.

Population	Wilcoxon Sign-Rank Test			Mode Shift ^a^
	IAM	TPM	SMM	
YS	0.125	0.844	0.990	L
EH	0.680	1.000	1.000	L
OS	0.125	0.973	0.973	L
SJ	0.578	1.000	1.000	L

Numbers in the table represent *p*-value; ^a^ normal l-shaped allele frequency distribution; IAM: the infinite allele model; SMM: stepwise mutation model; TPM: two-phase mutation model.

**Table 5:** Proportion of eight populations in each of the two inferred clusters.

Population	Inferred Cluster		Number of Individuals
	1	2	
YS	0.949	0.051	24
EH	0.084	0.916	24
OS	0.096	0.904	24
SJ	0.081	0.919	24

**Table 6:** Sampling sites, date of collection and sample size in the present study.

ID	Sampling Sites	Date	Number
YS	The Yellow Sea	January 2005	24
SJ	The Sea of Japan	September 2005	24
EH	Eastern Hokkaido	January 2004	24
OS	The Okhotsk Sea	April 2004	24
	Total	–	96

**Table 7:** Information for eight microsatellite loci and primers of Pacific cod in the present study.

Locus	Repeat Motif	Primer Sequence (5′–3′) (F, Forward; R, Reverse)	Size Range (bp)	GenBank No.
Gma102	(CTGT)_17_	F: TGGTTTCATTCGGTTTGGAT R: GGGCTCAGGTAAAGCCTCTT	221–275	DQ027808
Gma104	(GA)_8_(CAGAGACA) (GAGACA)_4_(GACA)_16_	F: AAAGAGAGCCACAGCCAGAT R: ATTCAACTGTTGGCCTCTGC	168–230	DQ027810
Gma107	(CTGT)_12_	F: GGGAGTGGAGTACAGGGTGA R: CCATTGTTTAACATCTGGGACA	195–243	DQ066622
Gma108	(GACA)_7_	F: AAGTCCCAACACACCAAAGC R: CTCCTCTCTCGCGCTCTTTA	210–280	DQ027813
Gma109	(GTCT)_7_G(GTCT)_18_	F: CATTTTACCTTTTGCTGAGGTG R: AAATTAAATTAGTTAGATGGAAAGA	257–373	DQ066623
Gmo08	(GACA)	F: GCAAAACGAGATGCACAGACACC R: TGGGGGAGGCATCTGTCATTCA	110–205	AFI159238
Gmo19	(GACA)	F: CACAGTGAAGTGAACCCACTG R: GTCTTGCCTGTAAGTCAGCTTG	120–220	AFI159232
Gmo37	(GACA)	F: GGCCAATGTTTCATAACTCT R: CGTGGGATACATGGGTACT	220–290	AFI159237
